# Predicting Atrial Fibrillation Ablation Outcomes: Machine Learning Model Development and Validation Using a Large Administrative Claims Database

**DOI:** 10.2196/77380

**Published:** 2025-12-31

**Authors:** Yijun Liu, Mustapha Oloko-Oba, Kathryn A Wood, Michael S Lloyd, Joyce C Ho, Vicki Stover Hertzberg

**Affiliations:** 1 Department of Data and Decision Sciences, Emory University, 36 Eagle Row, Atlanta, GA, 30322, United States, 1 4047275605; 2 Nell Hodgson Woodruff School of Nursing, Emory University, 1520 Clifton Road NE, Atlanta, GA, 30322, United States; 3 Division of Cardiology, Department of Medicine, Emory University, 100 Woodruff Circle, Atlanta, GA, 30322, United States; 4 Department of Computer Science, Emory University, 400 Dowman Drive, Atlanta, GA, 30322, United States

**Keywords:** administrative claims data, atrial fibrillation, atrial fibrillation ablation, machine learning, XGBoost, extreme gradient boosting

## Abstract

**Background:**

Atrial fibrillation (AF) ablation is an effective treatment for reducing episodes and improving quality of life in patients with AF. However, long-term AF-free rates after AF ablation are inconsistent across the population, ranging from 50% to 75%. Patient selection relies on individual clinical assessment, highlighting a critical gap in population-level predictive analytics. While existing risk scores (eg, CHADS_₂_ [congestive heart failure, hypertension, age ≥75 years, diabetes mellitus, and stroke], CHA_₂_DS_₂_-VASc [congestive heart failure, hypertension, age ≥75 years, diabetes mellitus, stroke, vascular disease, age, and sex category], CAAP-AF [coronary artery disease, left atrial diameter, age, AF, antiarrhythmic drugs, and female sex category]) have been applied to predict AF ablation outcomes, their performance in administrative claims data remains unclear. Leveraging large administrative claims databases represents an opportunity to develop standardized, scalable prediction models that could inform population health management and resource allocation at a national level.

**Objective:**

This study utilizes machine learning (ML) models on claims data to explore if integrating *International Classification of Diseases* (*ICD*) billing codes outperforms traditional stroke and AF risk scores in predicting 1-year AF ablation outcomes.

**Methods:**

We analyzed claims data from the Merative MarketScan Research Medicare database (2013‐2020) to identify 14,521 patients who underwent AF ablation. To predict 1-year AF-free outcomes, we developed logistic regression and extreme gradient boosting (XGBoost) models using demographic characteristics, comorbidity indices, and *ICD* diagnostic codes from the 2 years preceding ablation. Model predictions were compared with claims-based implementations of established risk scores—CHADS_2_, CHA_2_DS_2_-VASc, and a modified CAAP-AF (without left atrial diameter and the number of failed antiarrhythmic drugs). The ML models were also assessed on subgroups of patients with paroxysmal AF, persistent AF, and both AF and atrial flutter from October 2015 onward.

**Results:**

Among 14,521 patients (mean age 71.5, SD 5.31 y; n=5800, 39.94% female), AF ablation success occurred in 54.01% (n=7843). XGBoost achieved areas under the receiver operating characteristic curve (AUCs) of 0.528, 0.521, and 0.529 for the whole, female, and male AF ablation groups, respectively, and better discrimination than CHADS_2_, CHA_2_DS_2_-VASc, and the modified CAAP-AF in all AF ablation groups (whole population, female, and male). While CHA_2_DS_2_-VASc and the modified CAAP-AF showed higher recall (>0.798), their precision (<0.540) was lower than XGBoost (0.552‐0.556). In subgroup analyses of *International Classification of Disease, Tenth Revision* (*ICD-10*) patients (n=7646), the models incorporating *ICD* codes demonstrated better performance than those using only demographic and comorbidity data across most AF subtypes, with the highest AUC (0.544) observed in patients with paroxysmal AF.

**Conclusions:**

While the ML models achieved statistically significant improvements over claim-based implementations of established clinical risk scores (AUC 0.528‐0.544 vs 0.498‐0.505), the modest predictive performance highlights challenges in predicting procedural outcomes using administrative data that lack key clinical variables (eg, left atrial size and medication details). Our findings establish that while standardized outcome prediction using nationally available administrative data is technically feasible, current performance is insufficient for clinical decision-making and better suited for health system quality monitoring and comparative effectiveness research applications.

## Introduction

Although there is currently no cure for atrial fibrillation (AF), a major public health concern in the United States, AF ablation is the most effective treatment to restore normal sinus rhythm and decrease symptoms in episodes of paroxysmal or persistent AF, thereby reducing AF burden and improving quality of life [1-3]. AF is associated with an increased risk of cardiovascular events that may affect treatment outcomes. While various clinical risk factors are well understood, existing risk scores have shown inconsistent effectiveness in predicting AF ablation outcomes.

Existing risk scores, such as CHADS_2_ (congestive heart failure, hypertension, age ≥75 years, diabetes mellitus, and stroke) and CHA_2_DS_2_-VASc (congestive heart failure, hypertension, age ≥75 years, diabetes mellitus, stroke, vascular disease, age, and sex category), have traditionally been applied to predict stroke risk and are now also utilized in predicting outcomes following COVID-19, heart surgery, and AF ablation [4-11]. One risk prediction scale specifically designed to predict outcomes from AF ablation, the CAAP-AF (coronary artery disease, left atrial diameter, age, AF, antiarrhythmic drugs, and female sex category) risk score, addresses the presence or absence of coronary artery disease, the left atrial diameter, the presence of persistent AF, the number of antiarrhythmic drugs that have failed, and female sex [12].

Success rates for AF ablation from the literature vary based on individual clinical variables, such as the type of AF, left atrial size, or volume index [1,3,12,13], yet these variables are often difficult to access in large electronic health record (EHR) datasets. Patients can continue to experience episodes of AF following initial AF ablation with long-term AF-free rates after de novo AF ablation reported as 50%-75% [1,3,14]. Additionally, the chances of developing any complications after AF ablation range around 6%, with 0.1%-0.9% of patients experiencing complications that could result in death [15-17]. Given the modest success rates of AF ablation, the prediction of outcomes could be personalized to more easily identify those who would be most likely to benefit from AF ablation.

Machine learning (ML) has emerged as a powerful approach that leverages increased computational power with large datasets to help achieve complex decisions to guide clinical practice [18]. Artificial intelligence and ML have been used in the field of electrophysiology since the 1970s for automated electrocardiogram interpretation [18,19]. More recently, innovations in algorithms, development and labeling of large databases, and improvements in hardware and software have rapidly increased the role of ML in cardiac electrophysiology and cardiovascular imaging to identify predictors of patient outcomes [20]. Recent studies have demonstrated the potential for ML approaches in cardiovascular medicine, from achieving impressive diagnostic performance using novel data sources such as mobile phone acoustics for heart failure detection [21] to identifying practice gaps in stroke care guidelines [22] and showing predictive accuracy across various cardiovascular disease predictions [23]. ML has already been used to improve the prediction of AF ablation outcomes, primarily via EHRs. Nevertheless, health systems are not widely interoperable [24]; thus, extending these prognostic tools across multiple health systems is both costly and challenging. Studies utilizing EHR data have often been limited to datasets from 1 to 2 hospitals, limiting the generalizability of the models and hindering broad adoption [25,26].

Health insurance claims data, in comparison, are commonly collected, more readily available, and usually collected on a large national scale [27]. Although EHR data, which can include medications, laboratory data, and radiology reports, are more granular than claims data and can offer more accurate predictions, claims data’s breadth and consistency across health systems can potentially provide stronger external validity [28] and more cost-effective scaling. A recent study applied ML models on health insurance data for cardiovascular outcome prediction and achieved area under the receiver operating characteristic curve (AUC) of 0.68‐0.69 for heart failure readmission prediction [29], illustrating the potential for population-level insights using administrative databases. This wider coverage across patient populations and care settings may yield models that generalize more effectively, reducing the need for labor-intensive data extraction and curation that often blocks EHR-based projects. Furthermore, claims-based prognostic models can be used to enhance health care resource allocation by reducing unnecessary procedures in patients unlikely to benefit and increasing access to this effective therapy for appropriate candidates in resource-constrained regions. Thus, claims-based prognostic models represent a promising avenue for more accessible and large-scale prediction of AF ablation outcomes.

In this study, we propose to develop ML-based predictive models for outcomes of de novo AF ablation procedures using national-level claims data in the United States. Our goal is to evaluate an ML-derived risk prediction model for AF ablation patient outcomes. We hypothesize that ML models will be comparable to or exceed claim-based implementations of existing AF risk scores with respect to predictive power. Existing risk scores, including CHADS_2_ and CHA_2_DS_2_-VASc, have achieved nontrivial improvements in predicting the outcomes of AF procedures (AUCs of 0.785 and 0.830, respectively, in a dataset consisting of 565 patients) [28]. Thus, in this study, we utilize CHADS_2_ and CHA_2_DS_2_-VASc as a baseline to compare with our ML approaches. In addition, we also compare the performance between our ML models to a claims-based approximation of the CAAP-AF (modified CAAP-AF), a risk score specifically designed to predict AF ablation outcomes [12]. We also characterize outcomes by AF subtypes and sex and use different sets of parameters in the ML models to understand the contribution of individual factors to ML prediction performance.

## Methods

This research leveraged deidentified claims data sourced from the Merative MarketScan Research Medicare Databases (Merative, Inc.) between January 1, 2011, and December 31, 2021. MarketScan contains claims for individuals with Medicare Supplemental and Medicare Advantage plans.

### Patient Population

We analyzed Medicare claims data from January 1, 2011, to December 31, 2021, to identify patients who underwent AF ablation. Patients were included if they had a *Current Procedural Terminology* (*CPT*) code for AF ablation (93656) across either inpatient admission, inpatient services, and outpatient services tables in MarketScan. To ensure the accurate identification of AF ablation procedures, we required patients to have both *CPT* and a concurrent diagnosis of AF (*International Classification of Diseases, Ninth Revision* [*ICD-9*] code of “427.31” or *International Statistical Classification of Diseases, Tenth Revision* [*ICD-10*] code of “I48.X”). Each patient’s medical history included all *ICD* codes from visits within 2 years before the initial occurrence of AF ablation within our dataset. While the 2-year lookback period captures baseline characteristics, claims data do not allow definitive confirmation that these represent truly de novo ablations, as patients may have undergone prior ablations before their enrollment period or outside the MarketScan database. Therefore, our cohort represents the best approximation of first-time AF ablation procedures available from administrative claims data.

We focused exclusively on Medicare beneficiaries for several reasons. First, the MarketScan database maintains separate patient identifiers for Medicare and commercial claims datasets, preventing integration of these patients. Second, the typical age for the first AF ablation is between 55 and 62 years [1,2,13], which is commonly covered by Medicare. Moreover, the substantial absence of postoperative outcomes for patients in the commercial database rendered it unsuitable for this study. The final cohort included 14,521 Medicare patients.

### Outcome and Subgroup Definitions

Our study’s objective was to predict the binary outcome, success or failure, of AF ablation using patient demographics and prior medical history. Although the outpatient services table clearly documents the operation date for AF ablation, the inpatient admission and inpatient services tables only provide admission and discharge dates. To integrate the information across the 3 tables, we designated the admission date from the inpatient admission and inpatient services datasets as a surrogate for the AF ablation operation date in our analysis to maintain temporal coherence. Success was then defined as the absence of AF recurrence or repeat AF ablation between 6 and 12 months after the initial AF ablation procedure date, which is the standard interval before repeating an AF ablation according to current clinical practices [1,13]. To ensure the accurate identification of successful cases, we verified that all patients had at least 1 clinical follow-up visit within the first year after ablation.

The study also employed subgroup analysis by stratifying patients into 3 groups based on AF type: paroxysmal AF, persistent AF, and AF with flutter. This analysis was only possible after October 1, 2015, as *ICD-10* codes provide more detailed AF type distinctions compared to earlier *ICD-9* codes. We defined persistent AF as patients with *ICD-10* codes I48.1, I48.11, I48.19, I48.2, or I48.21. Note that this reflects current changes in the terminology of types of AF as it combines persistent AF and chronic AF. We defined paroxysmal AF as patients with *ICD-10* code of I48.0 or I48.20, and free of persistent AF. AF with atrial flutter were patients with any atrial flutter codes (*ICD-10*: I48.3 or I48.4).

### Data Processing

We constructed a comprehensive 2-year historical patient snapshot by linking records across the inpatient admission, inpatient services, and outpatient services tables using unique patient identifiers. For each patient, we extracted demographic variables (age, sex, region, and industry) at the time of the index ablation, along with the ablation date, failure date (if applicable), and all *ICD* codes within the 2 years preceding the index procedure. To standardize diagnostic codes across our study period, we used the *ICD-10* Lookup tool [30] to convert post-October 2015 *ICD-10* codes to their *ICD-9* equivalents. For computational efficiency and feature set manageability, we truncated all *ICD-9* codes to their first 3 digits, resulting in 785 *ICD* features and 19 demographic features. We used a binary measurement to denote whether or not a patient had the specific code within the 2 years prior to the initial ablation, thus avoiding extensive missing data.

We also calculated 2 established indices, the Charlson comorbidity index and the Elixhauser comorbidity index, to capture patients’ comorbid conditions [31,32]. These indices used a weighted system based on specific conditions to provide a score, with higher values indicating more severe comorbidities.

For the subgroup analysis, we utilized three distinct datasets: (1) all the simplified 3-digit *ICD* codes, demographic information, and 2 established indices; (2) demographic data and 2 established indices; and (3) solely demographic information.

### Modeling

We used 2 popular supervised ML classifiers: logistic regression and extreme gradient boosting (XGBoost) [33]. Logistic regression computes the probability of a binary outcome by employing a logistic function (sigmoid curve) to transform the linear combination of input features into probabilities. This model is particularly advantageous due to its simplicity and interpretability, especially in scenarios where the relationship between input variables and the outcome is expected to be linear. To tune the logistic regression model, we implemented grid search with 5-fold stratified cross-validation AUC as the primary evaluation metric. We explored regularization strengths (C values) on a logarithmic scale (0.001, 0.01, 0.1, 1, 10, 100) to address potential overfitting concerns. Both L1 (Lasso) and L2 (Ridge) regularization penalties were investigated to determine the optimal feature selection. We evaluated multiple solvers (“liblinear,” “lbfgs,” “newton-cg,” “sag,” “saga”) to identify the most computationally efficient optimization algorithm.

XGBoost represents a more sophisticated ML approach. XGBoost constructs multiple decision trees in a sequential manner, with each subsequent tree focusing on addressing the errors made by its predecessors. This method does not presuppose a linear relationship between input and output variables, offering greater flexibility and efficacy in dealing with larger and more intricate datasets. Despite its computational intensity, XGBoost is celebrated for its high efficiency and versatility, making it a potent tool in predictive modeling, especially in situations where the complexity of the data surpasses the capabilities of simpler models like logistic regression [27]. To tune the XGBoost hyperparameters, we implemented grid search with 5-fold stratified cross-validation with AUC as the primary evaluation metric. We explored a range of maximum depth values (3, 6, 9, 12, 15) to adequately capture complex feature interactions while avoiding overfitting. The learning rate varied across 0.01, 0.05, 0.1, and 0.2 to balance convergence speed and model accuracy, while the number of estimators was tested at 100, 200, 300, and 500 to determine the optimal number of boosting rounds.

The CHADS_2_ and CHA_2_DS_2_-VASc risk scores have been widely used to predict stroke risk in patients with AF [10,11,28]. CHADS_2_ is calculated using congestive heart failure, hypertension, age ≥75 years, diabetes, stroke (doubled), while CHA_2_DS_2_-VASc is computed using congestive heart failure, hypertension, age ≥75 (doubled) years, diabetes, stroke (doubled), vascular disease, age 65-74 years, and sex category (female). These risk scores more recently have been used to predict outcomes in patients with AF, heart failure, coronary artery disease, and postoperative AF undergoing cardiovascular surgical procedures [11,28]. CHADS_2_ and CHA_2_DS_2_-VASc risk scores were shown to be useful predictors of adverse events after AF ablation [10]. In addition to CHADS_2_ and CHA_2_DS_2_-VASc, we also evaluated a modified CAAP-AF, a risk score specifically designed to estimate the likelihood of remaining AF-free after ablation [12]. Due to the limitations of claims data, we only have information on coronary artery disease, age, AF type (persistent or longstanding, available only for patients after October 2015 using *ICD-10*), and sex. Left atrial diameter and the number of failed antiarrhythmic drugs are unavailable in MarketScan, which may impact our CAAP-AF comparison.

### Statistical Analysis

We compared patient characteristics between the groups using the Student *t* test for continuous variables and chi-square tests for categorical variables. Continuous variables were reported as mean (SD), while categorical variables were expressed as percentages.

We assessed the performance of the ML models and baseline risk scores using 5 metrics: AUC, area under the precision recall curve (AUPRC), precision, recall, and *F*_1_-score (the harmonic mean of precision and recall). Optimal hyperparameters for the ML models were first identified through 5-fold cross-validation on the full dataset. To measure performance, we then employed bootstrap resampling with 500 iterations. In each iteration, the ML model was trained on a bootstrap sample (drawn with replacement from the full dataset) using these optimal hyperparameters and then evaluated on the out-of-bag observations (samples not included in that bootstrap sample). This procedure was used to generate performance distributions and 95% CIs. Statistical significance was assessed using 1-tailed paired *t* tests on the bootstrap distributions to test whether ML outperformed the clinical scores (H₀: XGBoost≤clinical score).

### Ethical Considerations

This study used commercially available data that have been deidentified. As such, the study was deemed exempt by Emory University Institutional Review Board.

## Results

We leveraged the Merative MarketScan Research Medicare Databases (Merative, Inc.) between January 1, 2011, and December 31, 2021. To allow for a 2-year medical history and 1-year outcome assessment, the analytic cohort included patients observed between January 1, 2013, and December 31, 2020.

The demographic and clinical profiles of the patients with AF are detailed in Tables 1 and 2. Our study cohort consisted of 14,521 patients, with an average age of 71.5 years (SD 5.31). Successful outcomes from AF ablation procedures were observed in 54.01% (n=7843) of the patients. Female patients constituted 39.94% (n=5800) of the study population. Clinically, 24.73% (n=3591) of the patients were diagnosed with concomitant atrial flutter. As shown in Table 2, the Elixhauser comorbidity index showed limited variance, with 92.89% (n=13,488) of the patients in the “≥2” category. The precise identification of patients with paroxysmal and persistent AF was limited, relative to the total cohort, due to the use of *ICD-9* instead of *ICD-10* prior to October 2015. A total of 7646 patients were identified using *ICD-10* codes for AF ablation, demonstrating a slightly reduced AF ablation success rate of 53.28% in comparison to the broader patient population. A subset of 6983 patients was categorized as having paroxysmal or persistent AF. Within this subset, 37.63% (n=2877) were diagnosed with paroxysmal AF, while 53.70% (n=4106) had persistent AF. The AF ablation success rates for paroxysmal and persistent AF were 52.55% and 53.90%, respectively.

**Table 1.** Baseline demographic characteristics of patients undergoing AF^a^ ablation.^b^

Demographic variable	Overall (N=14,521)	AF ablation success (n=7843)	AF ablation failure (n=6678)	*P* value
Age (y), mean (SD)	71.5 (5.31)	71.5 (5.27)	71.6 (5.34)	.62
Female, n (%)	5800 (39.94)	3118 (39.76)	2682 (40.17)	.63
Region, n (%)				<.001
Northeast	2790 (19.21)	1544 (19.69)	1246 (18.66)	.12
North Central	4467 (30.76)	2263 (28.85)	2204 (33.00)	<.001
South	4733 (32.59)	2599 (33.14)	2134 (31.96)	.13
West	2393 (16.48)	1360 (17.34)	1033 (15.47)	.003
Unknown	138 (0.95)	77 (0.98)	61 (0.91)	.74
Industry, n (%)				<.001
Oil and gas extraction, mining	6 (0.04)	5 (0.06)	1 (0.01)	.30
Manufacturing, nondurable goods	3013 (20.75)	1486 (18.94)	1527 (22.87)	<.001
Manufacturing, durable goods	467 (3.21)	254 (3.24)	213 (3.19)	.91
Transportation, communication, utilities	1768 (12.18)	1007 (12.84)	761 (11.40)	.009
Retail trade	42 (0.29)	22 (0.28)	20 (0.30)	.95
Finance, insurance, real estate	661 (4.55)	371 (4.73)	290 (4.34)	.28
Services	2866 (19.74)	1479 (18.86)	1387 (20.77)	.004
Agriculture, forestry, fishing	4 (0.03)	2 (0.03)	2 (0.03)	>.99
Construction	33 (0.23)	20 (0.26)	13 (0.19)	.56
Wholesale	54 (0.37)	37 (0.47)	17 (0.25)	.05
Unknown	5607 (38.61)	3160 (40.29)	2447 (36.64)	<.001

a AF: atrial fibrillation.

b Industry is categorized based on the employer responsible for the claim payment, and regions follow the Census Bureau’s regional definitions.

**Table 2.** Baseline clinical characteristics of patients in sample undergoing AF^a^ ablation.^b^

Clinical variable	Overall (N=14,521), n (%)	AF ablation success (n=7843), n (%)	AF ablation failure (n=6678), n (%)	*P* value
Charlson comorbidity index				.29
0	4371 (30.10)	2375 (30.28)	1996 (29.89)	.61
1	4295 (29.58)	2277 (29.03)	2018 (30.22)	.87
≥2	5855 (40.32)	3191 (40.68)	2664 (39.89)	.61
Elixhauser comorbidity index				.45
0	44 (0.30)	24 (0.31)	20 (0.30)	>.99
1	989 (6.81)	515 (6.57)	474 (7.10)	>.99
≥2	13,488 (92.89)	7304 (93.13)	6184 (92.60)	>.99
Both atrial flutter and AF (*ICD-9*^c^ and *ICD-10*^d^)	3591 (24.73)	1963 (25.03)	1628 (24.38)	.38
Patients with *ICD-10*	7646 (52.65)	4074 (51.94)	3572 (53.49)	.51
Paroxysmal AF (*ICD-10* only)	2877 (37.63)	1512 (37.11)	1365 (38.21)	.33
Persistent AF (*ICD-10* only)	4106 (53.70)	2213 (54.32)	1893 (53.00)	.26
Unspecified AF	663 (8.67)	349 (8.57)	314 (8.79)	.76

a AF: atrial fibrillation.

b The paroxysmal and persistent AF only exists in the *ICD-10* space, of which the overall *ICD-10* population is 7646, success population is 4074, and failure population is 3572.

c *ICD-9*: *International Classification of Diseases, Ninth Revision*.

d *ICD-10*: *International Statistical Classification of Diseases, Tenth Revision*.

Table 3 shows the comparative performance of XGBoost, CHADS_2_, and CHA_2_DS_₂_-VASc of our entire study cohort. XGBoost consistently outperformed logistic regression in all analyses; therefore, only XGBoost results are presented for brevity. The full comparison between XGBoost and logistic regression is available in Multimedia Appendix 1. The XGBoost model exhibited modest predictive capability with an AUC of 0.528 for the overall population. It performed slightly better in male (AUC=0.529) than in female patients (AUC=0.521). The model achieved balanced performance with an *F*_1_-score of 0.581 and recall of 0.608, indicating that it captures most positive cases while maintaining reasonable precision at 0.556. Male patients showed slightly higher recall (0.614) than female patients (0.600).

**Table 3.** Performance comparison between XGBoost^a^ and CHADS_2_^b^ and CHA2_2_DS2_2_-VASc^c^ risk scores stratified by sex.

Metric	XGBoost^d^	CHADS_2_	CHA_2_DS_2_-VASc
Population (n=14,521)			
AUC^e^	0.528^f^ (0.519‐0.533)	0.498	0.498
AUPRC^g^	0.562^f^ (0.545‐0.578)	0.536	0.539
*F*_1_-score	0.581 (0.569‐0.594)	0.436	0.644
Precision	0.556^f^ (0.542‐0.570)	0.533	0.540
Recall	0.608 (0.585‐0.632)	0.368	0.799
Female (n=5800)			
AUC	0.521^f^ (0.510‐0.532)	0.498	0.500
AUPRC	0.558^f^ (0.533‐0.582)	0.536	0.538
*F*_1_-score	0.575 (0.556‐0.593)	0.436	0.698
Precision	0.552^f^ (0.530‐0.574)	0.533	0.538
Recall	0.600 (0.568‐0.632)	0.368	0.995
Male (n=8721)			
AUC	0.529^f^ (0.520‐0.539)	0.498	0.498
AUPRC	0.566^f^ (0.546‐0.588)	0.541	0.541
*F*_1_-score	0.585 (0.568‐0.601)	0.410	0.599
Precision	0.559^f^ (0.540‐0.578)	0.538	0.542
Recall	0.614 (0.582‐0.644)	0.331	0.669

a XGBoost: extreme gradient boosting.

b CHADS_2_: congestive heart failure, hypertension, age ≥75 years, diabetes mellitus, and stroke.

c CHA_2_DS_2_-VASc: congestive heart failure, hypertension, age ≥75 years, diabetes mellitus, stroke, vascular disease, age, and sex category.

d Cell values for XGBoost report average and the 95% CI in parentheses.

e AUC: area under the receiver operating characteristic curve.

f *P*<.001 (XGBoost vs both clinical scores).

g AUPRC: area under the precision recall curve.

Despite its moderate predictive power, the XGBoost model consistently outperformed both CHADS_2_ and CHA_2_DS_₂_-VASc scores across all patient cohorts in terms of AUC and AUPRC. Both risk scores (CHADS_2_ and CHA_₂_DS_₂_-VASc) performed poorly with AUC values worse than random chance (<0.5) except for CHA_2_DS_2_-VASc in the female subgroup (AUC=0.5). CHADS_2_ had poor recall (0.368) and low *F*_1_-scores (0.436), missing most positive cases. While CHA_2_DS_2_-VASc demonstrated high recall (0.799), particularly in female patients (0.995), the lower precision of 0.540 and AUC below 0.5 suggest that the score’s high sensitivity produces a higher false positive rate.

Table 4 presents a comparative analysis of the XGBoost and the modified CAAP-AF risk scores for the *ICD-10* cohort. XGBoost outperformed the modified CAAP-AF risk score with an overall AUC of 0.544 and AUPRC of 0.567 and consistent subgroup performance (female patients: AUC 0.543, AUPRC 0.569; male patients: AUC 0.545, AUPRC 0.567). In contrast, the modified CAAP-AF risk score achieved an overall AUC of 0.505, rising slightly to 0.511 in male patients but performing no better than random chance in female patients. While the modified CAAP-AF risk score exhibited high recall (0.999), capturing nearly all positive cases, it came at the cost of lower precision (0.533). XGBoost achieved better precision (0.552), albeit at a lower recall (0.793) and *F*_1_-score. All differences between the models were statistically significant.

**Table 4.** Performance comparison between XGBoost^a^ (ML^b^ model) and modified CAAP-AF^c^ risk score stratified by sex.

Metric	XGBoost^d^	Modified CAAP-AF
*ICD-10*^e^ population (n=7646)		
AUC^f^	0.544^g^ (0.535‐0.553)	0.505
AUPRC^h^	0.567^g^ (0.545‐0.590)	0.537
*F*_1_-score	0.651 (0.627‐0.672)	0.695^g^
Precision	0.552^g^ (0.535‐0.572)	0.533
Recall	0.793 (0.709‐0.867)	0.999^g^
Female (n=3161)		
AUC	0.543^g^ (0.516‐0.573)	0.500
AUPRC	0.569^g^ (0.533‐0.605)	0.533
*F*_1_-score	0.645 (0.615‐0.672)	0.694^g^
Precision	0.550^g^ (0.519‐0.578)	0.531
Recall	0.783 (0.701‐0.864)	1.000^g^
Male (n=4485)		
AUC	0.545^g^ (0.522‐0.569)	0.511
AUPRC	0.567^g^ (0.540‐0.596)	0.542
*F*_1_-score	0.655 (0.625‐0.678)	0.696^g^
Precision	0.554^g^ (0.533‐0.579)	0.535
Recall	0.801 (0.717‐0.875)	0.999^g^

a XGBoost: extreme gradient boosting.

b ML: machine learning.

c CAAP-AF: coronary artery disease, left atrial diameter, age, AF, antiarrhythmic drugs, and female sex category.

d Cell values for XGBoost report average and the 95% CI in parentheses.

e *ICD-10*: *International Statistical Classification of Diseases, Tenth Revision*.

f AUC: area under the receiver operating characteristic curve.

g *P*<.001 for comparison between XGBoost and modified CAAP-AF.

h AUPRC: area under the precision recall curve.

Table 5 presents the predictive model performance across atrial arrhythmia subgroups: paroxysmal AF, persistent AF, and AF with atrial flutter. A total of 3 feature sets were compared: *ICD* codes with demographics and comorbidity indices, demographics and comorbidity indices, and demographics only. On the entire *ICD-10* population, incorporating all the features (*ICD* codes with demographics and comorbidity indices) achieved the best performance across all 5 metrics when compared to the other 2 feature sets, with AUC of 0.544, AUPRC of 0.567, *F*_1_-score of 0.652, precision of 0.551, and recall of 0.798.

**Table 5.** Predictive performance by clinical and demographic predictors across atrial arrhythmia subgroups.^a^

Metric	*ICD*^b^+demographic+comorbidity indices, average (95% CI)	Demographic+comorbidity indices, average (95% CI)	Demographic only, average (95% CI)
Paroxysmal AF^c^ (n=2877)			
AUC^d^	0.538 (0.523‐0.553)	0.520 (0.530‐0.558)	0.532 (0.517‐0.547)
AUPRC^e^	0.557 (0.520‐0.596)	0.564 (0.529‐0.595)	0.547 (0.515‐0.582)
*F*_1_-score	0.563 (0.531‐0.593)	0.596 (0.540‐0.639)	0.645 (0.581‐0.680)
Precision	0.551 (0.514‐0.585)	0.548 (0.513‐0.582)	0.541 (0.509‐0.570)
Recall	0.576 (0.520‐0.629)	0.660 (0.525‐0.789)	0.808 (0.620‐0.948)
Persistent AF (n=4106)			
AUC	0.525 (0.512‐0.537)	0.518 (0.504‐0.531)	0.524 (0.510‐0.537)
AUPRC	0.561 (0.532‐0.592)	0.552 (0.522‐0.582)	0.557 (0.529‐0.586)
*F*_1_-score	0.575 (0.550‐0.596)	0.626 (0.586‐0.659)	0.658 (0.612‐0.689)
Precision	0.554 (0.525‐0.582)	0.549 (0.522‐0.574)	0.545 (0.520‐0.573)
Recall	0.598 (0.553‐0.641)	0.731 (0.622‐0.821)	0.834 (0.677‐0.947)
*ICD-10*^f^, with AF (n=1503)			
AUC	0.528 (0.506‐0.549)	0.514 (0.493‐0.535)	0.517 (0.497‐0.537)
AUPRC	0.564 (0.516‐0.609)	0.555 (0.512‐0.602)	0.558 (0.513‐0.605)
*F*_1_-score	0.600 (0.551‐0.644)	0.607 (0.552‐0.655)	0.611 (0.556‐0.655)
Precision	0.556 (0.508‐0.601)	0.547 (0.507‐0.589)	0.693 (0.548‐0.823)
Recall	0.657 (0.551‐0.770)	0.688 (0.552‐0.815)	0.550 (0.509‐0.597)
*ICD-10* population (n=7646)			
AUC	0.544 (0.535‐0.553)	0.533 (0.523‐0.542)	0.536 (0.528‐0.545)
AUPRC	0.567 (0.545‐0.589)	0.556 (0.532‐0.579)	0.559 (0.536‐0.581)
*F*_1_-score	0.652 (0.625‐0.672)	0.621 (0.595‐0.645)	0.645 (0.610‐0.672)
Precision	0.551 (0.531‐0.573)	0.550 (0.530‐0.570)	0.548 (0.528‐0.570)
Recall	0.798 (0.713‐0.871)	0.714 (0.644‐0.796)	0.787 (0.681‐0.878)

a This population only includes patients who had their first atrial fibrillation ablation in or after October 2015. Predictors include *ICD* codes of patients’ past medical history and demographic variables (region, sex, age, and industry).

b *ICD*: *International Classification of Diseases*.

c AF: atrial fibrillation.

d AUC: area under the receiver operating characteristic curve.

e AUPRC: area under the precision recall curve.

f *ICD-10*: *International Statistical Classification of Diseases, Tenth Revision*.

Within the atrial arrhythmia subgroups, models incorporating all features consistently achieved the highest AUC and AUPRC across all 3 subgroups. However, performance patterns for *F*_1_-score and recall varied by subgroup. For paroxysmal AF and persistent AF, the full model also achieved the highest precision (0.551 and 0.554, respectively), but the models with demographics only had better recall (0.808 and 0.834, respectively) and *F*_1_-scores (0.645 and 0.658, respectively). For AF with atrial flutter, the model with demographics only achieved the highest *F*_1_-score (0.611) and precision (0.693), whereas the model with demographics and comorbidity indices achieved the highest recall (0.688).

## Discussion

### Principal Findings

In this study, we developed ML models that predict the outcomes of de novo AF ablation procedures. Our XGBoost model demonstrated statistically significant improved performance compared to 3 different claim-based implementations of clinical risk scores (CHADS_2_, CHA_2_DS_2_-VASc, and a limited, modified CAAP-AF without left atrial diameter and the number of failed antiarrhythmic drugs) in all patient and sex subgroups in terms of AUC and AUPRC. While the 2 risk scores achieved higher recall than XGBoost, they demonstrated lower precision and weaker discrimination (near random chance). However, XGBoost’s predictive ability for outcomes after AF ablation was found to be lower in female patients than it was in male patients or in the entire population. There was no difference in AUC when comparing CHADS_2_ to CHA_2_DS_2_-VASc risk scores for outcomes after AF ablation except for female patients, where CHA_2_DS_2_-VASc performs better than CHADS_2_.

When comparing outcomes across different AF subtypes (paroxysmal, persistent, or AF with atrial flutter), we observed heterogeneous patterns in the value of adding *ICD* code features. For persistent AF and AF with atrial flutter, the models incorporating *ICD* code features demonstrated superior discriminative power (AUC and AUPRC) compared to models using either demographic or clinical variables alone or those combined with comorbidity indices (Charlson comorbidity index and the Elixhauser comorbidity index). However, in the paroxysmal AF subgroup, the model using only demographics and comorbidity indices slightly outperformed the full model with *ICD* codes in terms of AUPRC but not AUC. Additionally, models using demographics only consistently achieved higher recall across all subgroups at the expense of lower precision and overall discriminative performance (AUC and AUPRC), revealing a trade-off between sensitivity and specificity in feature selection. The use of these ML models may be useful in clinical practice in patient selection for AF ablation in the future.

### Comparison to Prior Work

Claims data present challenges for outcome prediction, despite being readily available. Previous clinical models for predicting AF ablation success have reported an AUC ranging from 0.55 to 0.65, with only 3 models achieving an AUC of 0.75 [4,5,12]. In other studies, CHADS_2_ and CHA_2_DS_2_-VASc achieved an AUC of 0.785 and 0.830, respectively, in predicting complications after AF ablation [6]. However, in our study, CHADS_2_ and CHA_2_DS_2_-VASc only achieved an AUC of 0.498‐0.5, performing almost worse than random guessing. It is important to note that while CHADS_2_ and CHA_2_DS_2_-VASc have been used for predicting procedural outcomes [4-6], they were originally designed to estimate stroke risk rather than ablation recurrence, and thus their lower performance in this study potentially reflects use outside of the intended purpose rather than a failure of the scores themselves.

The modified CAAP-AF reached AUC greater than 0.650 [12] with the data from its original study, yet in our implementation, it achieved no better than 0.511. However, the CAAP-AF score used in our study was a modified, claims-based approximation that excluded left atrial diameter and the number of failed antiarrhythmic drugs, as these are not available in claims data. Therefore, our comparison does not represent a true head-to-head evaluation of the original CAAP-AF model, and the ML model’s advantage should be interpreted with this limitation in mind.

These findings highlight the significant difficulty in predicting AF ablation success and failure using claims data, reflecting broader challenges in health care outcome prediction where administrative databases consistently underperform compared to clinical models due to the absence of key physiological and procedural variables, a pattern observed across multiple medical specialties and intervention types [34,35]. In contrast, our ML models achieved AUCs of 0.521‐0.529, showing marginal improvement.

Despite the modest predictive performance of the ML models, our claims-based approach has significant potential for standardization across health care systems, as it relies on widely used *ICD* and *CPT* coding systems rather than institution-specific EHR implementations. However, adoption barriers remain, including variations in coding practices across institutions, the challenge of integrating predictive tools into clinical workflows, and potential resistance from clinicians who may prioritize clinical judgment over algorithmic recommendations. Given the relatively low AUC values observed, these models should be viewed as a foundational step toward using claims data to predict the outcomes of AF ablation procedures, rather than as tools ready for clinical deployment.

Beyond demonstrating that ML models outperform traditional risk scores, we conducted an analysis to understand what types of features should be included in the ML models across clinically relevant AF subgroups. We evaluated three feature sets: (1) demographic information alone; (2) demographics plus comorbidity indices; and (3) the full features incorporating *ICD* codes, demographics, and comorbidity indices. These were tested across 3 clinically distinct subgroups (paroxysmal AF, persistent AF, and AF with atrial flutter) identifiable only through *ICD-10* coding, yielding 16 unique ML models. Across persistent AF and AF with atrial flutter subgroups, ML models performed best when including *ICD* codes as features, highlighting the importance of diagnostic coding data. Among the 3 subgroups (paroxysmal AF, persistent AF, and patients with atrial flutter), the ML models performed best for patients with paroxysmal AF, and patients with persistent AF had the least success. The entire *ICD-10* population achieved the highest overall AUC compared to other subgroups, which was likely due to the larger sample size.

### Future Directions

Our findings demonstrate that ML models using *ICD* codes to estimate AF ablation procedural outcomes are robust and valid across populations. However, the model’s current predictive power in this study remains insufficient for clinical decision-making. Improvement of outcome predictions for AF ablation using ML has the potential for widespread use in research and clinical practice to determine optimal patient selection for AF ablation and the management of patients with AF. Advances in artificial intelligence and ML technology have an ability to rapidly analyze and synthesize innumerable variables to predict outcomes of AF ablation and discover new patterns of clinical variables that greatly surpass prior conventional methods of gaging success. These findings will be important to consider, as health care policymakers struggle to allocate limited resources to as many patients as possible and search for ways to improve patient outcomes. ML technologies will play increasingly more important roles in medicine with future advances as we better learn how to incorporate ML for better health care resource allocation as well as improvements in clinical practice and patient outcomes.

Several specific clinical implementation scenarios could leverage these predictive tools to enhance AF ablation care delivery. An important deployment consideration is the metric to optimize, as our findings revealed a trade-off between precision and recall. For population health monitoring, quality improvement initiatives, or patient counseling, high-recall models may be preferred. Conversely, for resource allocation decisions such as prioritizing ablation slots during periods of limited procedure capacity, high-precision models would be more appropriate to minimize false positives. Clinicians could use model predictions to provide patients with more personalized success probability estimates during shared decision-making discussions, helping patients make more informed treatment choices. Alternatively, these models could guide the development of alternative treatment pathways or enhanced monitoring protocols for patients with consistently lower predicted success rates. Future research should focus on developing implementation frameworks that appropriately balance algorithmic predictions with clinical judgment and metric selection based on clinical context while ensuring equitable access to AF ablation across diverse patient populations.

### Limitations

First, our study relied exclusively on Medicare Advantage and Medicare Supplemental claims, which skews the cohort toward older patients. Although first ablations often occur between ages 55‐62 years, our findings may not be generalizable to younger populations with different comorbidity profiles and procedural outcomes. The etiology and pathophysiology of AF may differ between younger and older patients, which could affect both the predictive variables and outcomes in ways that our models may not capture. Future work should validate and potentially recalibrate these models in younger and more diverse populations to ensure broader clinical utility.

Second, as with all administrative data, coding errors and inconsistencies are possible. We mitigated this by truncating *ICD* codes into broader categories, incorporating 2 established comorbidity indices (Charlson comorbidity index and the Elixhauser comorbidity index), and requiring that all patients had a documented AF diagnosis before ablation. Despite these steps, misclassification could still reduce model performance. Moreover, truncating *ICD-9* codes to the first 3 digits may also have reduced diagnostic specificity. This limitation may explain our unexpected finding that the model using only demographics and comorbidity indices slightly outperformed the full model with *ICD* codes in the paroxysmal AF subgroup. The truncated *ICD* codes may have introduced noise rather than signal for this subgroup, particularly if patients with paroxysmal AF have less diverse billing code profiles making the additional *ICD* code features less informative. Future analysis may mitigate the issue by integrating claims with richer data sources to cross-validate the information.

Third, while we aimed to study de novo AF ablation procedures, administrative claims data have inherent limitations in both identifying first-time ablations and measuring their outcomes. Although we identified the initial occurrence of AF ablation within our dataset, we cannot definitively exclude patients who may have undergone prior ablations before their enrollment in the database or at facilities not captured in MarketScan. Furthermore, our outcome definition may be subject to misclassification as we are using billing codes as a proxy for clinical recurrence. Asymptomatic or unrecorded recurrences could be missed (falsely classified as success), while unrelated visits coded with previous AF could be incorrectly classified as failures. Additionally, patients with undetected prior ablations may have different recurrence trajectories than true first-time procedures, further complicating outcome assessment. AF recurrence is best confirmed with secondary data sources such as Holter monitoring or electrocardiogram data.

Fourth, claims data lack important clinical variables known to influence AF ablation outcomes, such as left atrial size, ejection fraction, specific antiarrhythmic medications, and procedural details (catheter type, ablation strategy). This limitation likely contributed to our models’ modest predictive performance compared to clinical prediction models that incorporate these variables. Additionally, the limited variance in the Elixhauser comorbidity index, where 92.89% (n=13,488) of patients fell into a single category (≥2), reduced its discriminative value and may explain why adding comorbidity indices to demographic variables resulted in minimal or slightly negative effects on model performance in some subgroups. While we cannot address this limitation within our study design, future research could explore hybrid approaches that combine claims data with targeted clinical data collection for key predictive variables. However, we note this may limit the scalability and standardization advantages that motivated our claims-based approach.

Finally, given the proprietary nature of MarketScan data, direct replication is constrained. To enhance transparency and reproducibility, we documented our data source, inclusion and exclusion criteria, billing codes, and potential confounders and released the analytic code in a public GitHub repository to facilitate replication [36]. This enables researchers with access to similar claims databases to replicate our methodology, though exact replication would require the same data source.

### Conclusions

In this study, we developed and evaluated ML models using MarketScan claims data to predict 1-year AF ablation outcomes. Across the overall cohort and sex-stratified groups, ML models modestly but consistently outperformed claim-based implementations of established clinical risk scores. In the *ICD-10* subset, incorporating *ICD* diagnostic codes improved performance relative to the models using only demographic and comorbidity indices over most subgroups. Our findings demonstrate the limitations of ML approaches when applied to claims data that lack key clinical variables, such as left atrial size, ejection fraction, and medication details. The modest predictive performance indicates that current claims-based models are insufficient for individual clinical decision-making. Despite these constraints, our work establishes that standardized, population-level outcome prediction using nationally available administrative data is technically feasible, providing capability that could complement existing clinical tools for health system quality monitoring and research applications. These results contribute important insights into the potential and limitations of claims-based prediction models for population-level analyses and comparative effectiveness research.

## Supplementary material

10.2196/77380 Multimedia Appendix 1 Logistic regression results.

## Abbreviations

AF: atrial fibrillation
AUC: area under the receiver operating characteristic curve
AUPRC: area under the precision recall curve
CAAP-AF: coronary artery disease, left atrial diameter, age, AF, antiarrhythmic drugs, and female sex category
CHA_2_DS_2_-VASc: congestive heart failure, hypertension, age ≥75 years, diabetes mellitus, stroke, vascular disease, age, and sex category
CHADS_2_: congestive heart failure, hypertension, age ≥75 years, diabetes mellitus, and stroke
*CPT*: *Current Procedural Terminology*
EHR: electronic health record
*ICD*: *International Classification of Disease*
*ICD-10*: *International Statistical Classification of Diseases, Tenth Revision*
*ICD-9*: *International Classification of Diseases, Ninth Revision*
ML: machine learning
XGBoost: extreme gradient boosting
