# Supplementary material for: Predicting Atrial Fibrillation Ablation Outcomes: Machine Learning Model Development and Validation Using a Large Administrative Claims Database
Source: JMIR Cardio. 2025 Dec 31;9:e77380. doi: 10.2196/77380 (PMC12755845; doi:10.2196/77380)
Supplement: Multimedia Appendix 1 [file cardio-v9-e77380-s001.docx]

## Multimedia Appendix 1.

**Table S1.** Area under the receiver operating characteristic curve (AUC) Performance for Logistic regressions on total population and ICD-10 Population

|  | Logistic Regression | XGBoost |
| --- | --- | --- |
| Population (n=14,521) | 0.505 | 0.528 |
| Female (n=5,800) | 0.509 | 0.521 |
| Male (n=8,721) | 0.501 | 0.529 |
| ICD-10 Population (n=7,646) | 0.515 | 0.544 |
| Female (n=3,161) | 0.521 | 0.543 |
| Male (n=4,485) | 0.511 | 0.545 |

**Table S2.** AUC Performance of Logistic Regression on the different feature sets.

|  | **ICD + Demographic Characteristics + Comorbidity Indices** | **Demographic Characteristics + Comorbidity Indices** | **Demographic Characteristics** |
| --- | --- | --- | --- |
| **Paroxysmal AF**  **(n=2,877)** | 0.493 | 0.544 | 0.508 |
| **Persistent AF**  **(n=4,106)** | 0.505 | 0.513 | 0.522 |
| **ICD-10, With Atrial Flutter**  **(n=1,503)** | 0.5 | 0.5 | 0.498 |
| **ICD-10 Population**  **(n=7,646)** | 0.520 | 0.512 | 0.530 |
